# Robust Vehicle Detection in Aerial Images Based on Cascaded Convolutional Neural Networks

**DOI:** 10.3390/s17122720

**Published:** 2017-11-24

**Authors:** Jiandan Zhong, Tao Lei, Guangle Yao

**Affiliations:** 1 Institute of Optics and Electronics, Chinese Academy of Sciences, No. 1, Guangdian Avenue, Chengdu 610209, China; taoleiyan@ioe.ac.cn (T.L.); guangle.yao@std.uestc.edu.cn (G.Y.); 2 School of Optoelectronic Information, University of Electronic Science and Technology of China, No. 4, Section 2, North Jianshe Road, Chengdu 610054, China; 3 University of Chinese Academy of Sciences, 19 A Yuquan Rd, Shijingshan District, Beijing 100039, China

**Keywords:** vehicle detection, convolutional neural network, aerial image, deep learning

## Abstract

Vehicle detection in aerial images is an important and challenging task. Traditionally, many target detection models based on sliding-window fashion were developed and achieved acceptable performance, but these models are time-consuming in the detection phase. Recently, with the great success of convolutional neural networks (CNNs) in computer vision, many state-of-the-art detectors have been designed based on deep CNNs. However, these CNN-based detectors are inefficient when applied in aerial image data due to the fact that the existing CNN-based models struggle with small-size object detection and precise localization. To improve the detection accuracy without decreasing speed, we propose a CNN-based detection model combining two independent convolutional neural networks, where the first network is applied to generate a set of vehicle-like regions from multi-feature maps of different hierarchies and scales. Because the multi-feature maps combine the advantage of the deep and shallow convolutional layer, the first network performs well on locating the small targets in aerial image data. Then, the generated candidate regions are fed into the second network for feature extraction and decision making. Comprehensive experiments are conducted on the Vehicle Detection in Aerial Imagery (VEDAI) dataset and Munich vehicle dataset. The proposed cascaded detection model yields high performance, not only in detection accuracy but also in detection speed.

## 1. Introduction

Vehicle detection in aerial images is an important task in various fields, such as: remote sensing, intelligent transportation and military reconnaissance. With the great development of Unmanned Aerial Vehicle (UAV) technologies, aerial images are captured conveniently and flexibly in this way. For the growing aerial imagery data, vehicle detection has become a challenge, attracting extensive attention recently. As a fundamental task in computer vision, vehicle detection is widely studied in some practical applications, such as traffic monitoring [1,2] and safety assistant driving [3,4], but for aerial images, it is still a tough problem due to the obscurity, relatively small size of the targets and cluttered backgrounds. Additionally, other objects such as big containers and road marks always show a similar appearance to vehicles, which will cause false detection or accuracy loss. Furthermore, in a detection model, not only detection accuracy is demanded, but also good detection speed.

In last decade, target detection technology has developed greatly, and can be roughly divided into three stages. In the first stage, the combination of hand-crafted features and discriminative classifiers were utilized to detect targets. On the one hand, some classical method like Histogram of Oriented Gradient (HOG) [5] and Scale-Invariant Feature Transform (SIFT) [6] were designed for feature extraction. On the other hand, the discriminative classifiers like Support Vector Machine (SVM) [7] and Ada-Boost [8] were adopted for classification. Felzenszwalb et al. [9] proposed a deformable parts model (DPM), which employs various trained components to detect targets from an image pyramid in sliding-window fashion. Although DPM is an excellent detector, the sliding-window strategy is time consuming in the detection phase. In the second stage, the sliding-window method was replaced with a region proposal way [10,11,12]. It means that the detectors don’t need to detect the targets from the image pyramid, but from thousands of candidate target-like regions. This is a very efficient way to reduce the detection time. For example, the candidate regions of an image (of size 400 × 500) is about 10^3^, which is much less than the search space (about 10^4^~10^5^) of the image pyramid with a sliding-window way. The third stage started in 2012, when Krizhevsky et al. [13] applied the convolutional neural networks (CNNs) method in an image classification challenge (ILSVRC2012) and obtained striking results [14], which turned CNN-based methods into the mainstream in the field of computer vision. Recently, Girshick et al. [15] and Sermanet et al. [16] proposed efficient detection models based on CNNs. Especially, the method described in [15], called Regions with CNN (R-CNN), has become the baseline for the detection framework. The workflow of R-CNN is mainly divided into two steps: (a) it employs the region proposal method discussed in [10] to generate a set of candidate regions, and then (b) these regions are warped into a fixed size and fed into a CNN to extract the deep features. From the extensive experimental results, the CNN features show more discriminative capability than the traditional hand-crafted features. It is noteworthy that the region proposal method [10] always takes several seconds on an image of medium size (e.g., 500 × 300 pixels), and the CNN features in the different regions would be extracted repeatedly. Then, improved methods named SPP-Net [17] and Fast R-CNN [18] were proposed to accelerate the detection speed. In Fast R-CNN, a region of interesting (ROI) strategy was used to deal with the problem of repeated CNN feature extraction, which speeds up the CNN feature extraction procedure significantly. Another main bottleneck of R-CNN is the computational costs in the region proposal procedure. Ren et al. [19] proposed a CNN-based architecture called Region Proposal Network (RPN) to replace the method described in [10]. They combined RPN with Fast R-CNN and trained a unified detection model, which achieves state-of-the-art performance on PASCAL 2007/2012 and MS COCO datasets [20]. The detection speed reached 5 fps with a VGG-16 network [21].

Although the works [17,18,19] show promising results in target detection, they are not suitable for aerial images. The first reason is that the vehicles in the aerial image are relatively small in size (the average size of a vehicle is 40 × 20 pixels), and due to the coarseness of the feature map (output of the deep convolutional layer of the CNN), RPN has the poor localization performance for small targets. Moreover, the detection models [17,18,19] are designed for multi-category detection, but for the specific category “vehicle”, they perform poorly due to the false positives. The second reason is that the vehicles always appear as vehicle roofs in aerial images, which has similarity with other background targets. This would cause accuracy loss without specific training. Furthermore, unlike the large scale public datasets (such as: ImageNet [14] and MS COCO [20]) comprising millions of images, the training data of available annotated aerial image datasets (for vehicles) is insufficient.

**Figure 1:**
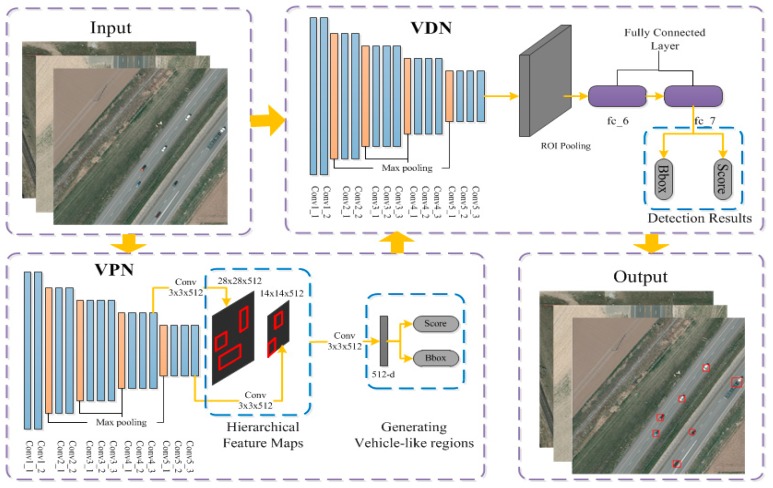
Framework of the proposed model.

In this paper, we propose a cascaded CNN model to detect vehicles in aerial imagery data, which maintains high detection accuracy and fast speed. The framework of our model, shown in Figure 1, comprises two CNN-based networks. The first network is called the vehicle-regions proposal network (VPN) which aims to generate the vehicle-like regions. The second part is the vehicle detection network (VDN) which performs decision making for the regions generated by the first network. The workflow of the detection phase is divided into three steps: (1) an input image is put into the VPN to generate candidate regions, (2) the generated regions incorporating the input image are fed into VDN to extract each region’s feature and predict the confidence score, (3) the regions with high score (greater than a threshold) are output as detections. Compared with the work [19], our model has three main differences: (1) unlike the work [19] that trains a unified network, we train two independent networks. It means that we do not share the convolutional layers of two networks, which avoids re-training the unshared layers of two networks; (2) the feature maps output from the deep convolutional layers (of CNN) can detect the target with high recall but poor localization performance, while the feature map from the shallow layers have better localization performance but obtain a reduced recall [22]. To take advantage of both, we combine the feature maps of the shallow layers and deep layers together to generate the vehicle-like regions in various scales and hierarchies. In this way, our method obtains finer and more accurate vehicle-like regions than RPN; (3) the VDN is trained as a specific category detector which is applied to detecting multi-type vehicles.

Additionally, the original annotations of aerial image data are not suitable for VDN due to the fact that the bounding boxes of targets are annotated with various orientations. In this paper, the target bounding box is transformed into a vertical or horizontal format. To avoid overfitting in such a deep network, the training data are augmented by flipping and rotating operations.

The contributions of this paper are:

- A fast and accurate detection model is designed for vehicle detection in aerial images, which is different from the traditional sliding-window-based model and the recent CNN-based model. Our model is a cascaded architecture which incorporates two independent CNNs: the first is employed to generate vehicle-like regions, and the second is a specific-category detector which makes a final decision.
- The VPN is proposed to extract vehicle-like regions. Unlike the RPN that uses only one feature map, the proposed VPN combines multi-feature maps of different size and hierarchy for generating better vehicle-like regions. Actually, the proposed VPN takes effect on other categories as well, especially for the small targets in aerial image.
- A category-specific detector named VDN is developed, which can detect the various types of vehicles in aerial images. Additionally, unlike the Faster R-CNN which employs two-stage alternative training to share some convolutional layers, our VDN and VPN are trained independently once to increase training efficiency. This also avoids re-training the unshared layers that exist in the two networks. The VDN can be easily transferred to other target detection tasks.
- An augmented dataset is built for vehicle detection in aerial images. To make the training data fit for our CNN-based model, we re-annotated the available public dataset. To avoid the overfitting, we performed data augmentation in two operations.

The rest of this paper is organized as follows: in Section 2, we describe the related work about the region proposal method, CNN-based detectors and the related detectors designed for aerial image data. The preliminary theories and analysis of data augmentation, VPN and VDN are introduced in Section 3. In Section 4, we show the evaluation results on the VEADI and Munich vehicle datasets. We conclude this paper and propose some future work in Section 5.

## 2. Related Work

In this section we review the recent methodologies related to target detection. Moreover, some recent vehicle detection methods are introduced as well.

### 2.1. Region Proposal Method

In many target detection approaches a small number of candidate regions which cover all the objects in an image is proposed, and extensive studies on region proposal methods can be found in [10,11,12,19,23,24,25,26,27,28]. Carreira et al. [23] proposed a rough segmentation method to generate candidate regions, which has been shown to be effective. Promising results were obtained by the method of estimating the objectness score on an image [11,12,24]. Uijlings et al. [10] proposed the Selective Search (SS) way, which generates regions with better objectness based on its hierarchical segmentation and grouping strategies. Additionally, the works [29,30,31,32] adopted the method of super-pixels segmentation to generate image regions. In particular, Achanta et al. [29] proposed a simple and efficient method called simple linear iterative clustering (SLIC), which performs well in image segmentation. However, the candidate regions generated by a segmentation method cannot be directly fed into CNN for feature extraction because the segmented regions are polygonal regions which should be converted into rectangles first. Recently, the use of CNN-based methods to generate the candidate regions has become a trend. Deepbox [28] trained a slight CNN model and learned to re-rank candidate regions generated by [24]. Ren et al. employed RPN [19] and Fast R-CNN [18] to train a unified detection model. Through this two-stage alternative training, this model yields state-of-the-art performance.

### 2.2. Target Detection with the CNN-Based Models

By virtue of its powerful feature extraction capability, CNN has been widely used in target detection. References [15,16,33] are the pioneering works of employing CNN to deal with target detection tasks. Although these works perform well in detection accuracy, they are time consuming in feature extraction. Then, shared convolution computation has attracted more attention, and the methods in references [17,18,34] were proposed to deal with this problem. The SPP-Net [17] and Fast R-CNN [18] proposed further improvements on [15], which showed compelling accuracy and speed. To obtain more efficient and accurate localization of targets, more and more works have employed CNN-based models to generate proposal regions. RPN [19] and MultiBox [35] are two representative works. Moreover, Redmon et al. [36] presented a proposal-free framework named You Only Look Once (YOLO), which directly predicts bounding boxes and evaluate probabilities without proposing candidate regions. In practice, the region-based models like [19] outperform YOLO with respect to the detection accuracy.

### 2.3. Vehicle Detection in Aerial Imagery

Detecting vehicles in aerial imagery data is an interesting topic nowadays. Xu et al. [37] proposed a hybrid method which adopted the traditional hand-crafted features (HOG) and linear SVM. For vehicles on a highway, this method yields high performance, however, this method uses a lot of road-line information as auxiliary. Nassim et al. [38] proposed a deep learning method to detect vehicles in the aerial images captured by UAVs, where they first segment the regions of interest in the image and then feed them into a CNN model for feature extraction. The final decision was made by a SVM. Qu et al. [39] combined the region proposal method in [11] with SPP-Net [17] to build a vehicle detection model. The works [38,39] both employed the CNN as a feature extractor. Tang et al. [40] proposed the Hyper Region Proposal Network (HRPN) to localize the vehicle-like regions, and utilized hard negative examples to improve the detection accuracy. Deng et al. [41] modified RPN and Fast R-CNN to build a unified CNN-based model for vehicle detection. In fact, the works [40,41] concatenated multiple convolution layers into one hyper-feature map, but the multi-hierarchy and scale information concepts were not adopted.

## 3. Overview of the Proposed Model

The proposed vehicle detection model (shown in Figure 1) consists of two cascaded CNNs: a vehicle-regions proposal network (VPN) and a vehicle detection network (VDN), which are trained independently during the training phase. The VPN aims to generate candidate vehicle-like regions accurately at first. Afterwards, these vehicle-like regions are fed into the VDN to make inference. Moreover, to avoid overfitting, we augment the original dataset artificially.

### 3.1. Training Data Augmentation

The VEDAI [42] and Munich vehicle datasets [43] are adopt to evaluate the performance of detection model. The VEDAI dataset includes about 1240 images with two kinds of resolutions: 1024 × 1024 and 512 × 512 pixels. The training data is relatively small, which is just applicable for many situations (such as vehicle detection in urban, country road, crop and residential areas) and may not be able to meet the needs of a larger range practical application (e.g., to detect the vehicles are partially covered by vehicle-like regions with trees or artificial structures). Therefore, the experimental design of this study makes it difficult to comment on the feasibility of large-scale implementation. Additionally, it is very inefficient to directly use CNN-based models for target detection in the image with such a large scale of resolution (5616 × 3744 pixels). For this reason an input image will be resized by the designed CNN model (the shorter side of the image will be resized to 600 pixels for convenience). For large size images, this will cause an accuracy loss. Hence, the images in Munich vehicle dataset are cropped to a size of 702 × 468 pixels for training and testing.

**Figure 2:**
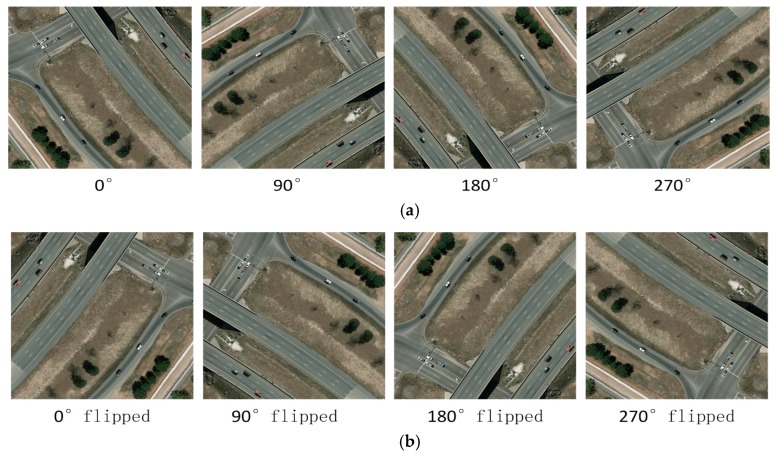
(**a**) Training images are rotated with four angles in clockwise; (**b**) The flip operation of the training images.

Additionally, due to the lack of training data, we augment the training data by two operations: rotation and flip (described in Figure 2). For each training image, we rotate it with four angles (0°, 90°, 180° and 270°) in a clockwise direction. Further, we flip the rotated images as well (shown in Figure 2b). Another problem is that the original annotation information of these datasets is not suitable for CNN-based models, because the bounding boxes of targets are rotated with various angles. We adjust the coordinates of bounding box according to the steps below:

(1) Obtaining the original four coordinates of bounding box: [*x_lt_*, *y_lt_*], [*x_rt_*, *y_rt_*], [*x_rb_*, *y_rb_*] and [*x_lb_*, *y_lb_*];
(2) Calculating the *height*: *h* = *max*(*y_lt_*, *y_rt_*, *y_rb_*, *y_lb_*) − *min*(*y_lt_*, *y_rt_*, *y_rb_*, *y_lb_*);
(3) Calculating the *width*: *w* = *max*(*x_lt_*, *x_rt_*, *x_rb_*, *x_lb_*) − *min*(*x_lt_*, *x_rt_*, *x_rb_*, *x_lb_*);
(4) Updating the left-top coordinate as [*min*(*x_lt_*, *x_rt_*, *x_rb_*, *x_lb_*), *min*(*y_lt_*, *y_rt_*, *y_rb_*, *y_lb_*)];
(5) Using the height, width and left-top coordinate to update other coordinates.

**Figure 3:**
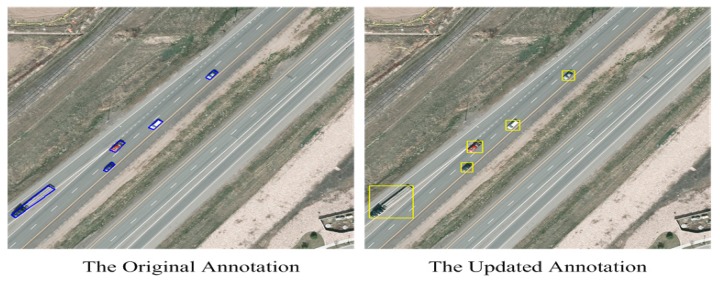
Examples of the original and updated annotations.

Figure 3 gives examples of the original annotation and the updated annotation.

### 3.2. Vehicle-Regions Proposal Network

The proposed VPN takes an image as input and outputs a set of vehicle-like regions with the corresponding objectness scores. RPN [19] adopts the feature map of the deep convolutional layer to generate candidate regions. To improve this framework, references [41,44] concatenated multiple convolutional layers and built a hyper-feature map. Enlightened by these works [19,41,44], we combine deep and shallow convolutional layers to construct a hierarchical structure which comprises coarse and fine feature maps with various sizes and scales. In our VPN, the region proposals are generated from each feature map. As a result, more accurate regions are proposed than by using the methods of [19,41,44], which adopt only one feature map. The detailed description of VPN is provided below.

#### 3.2.1. Overview of the Architecture

**Figure 4:**
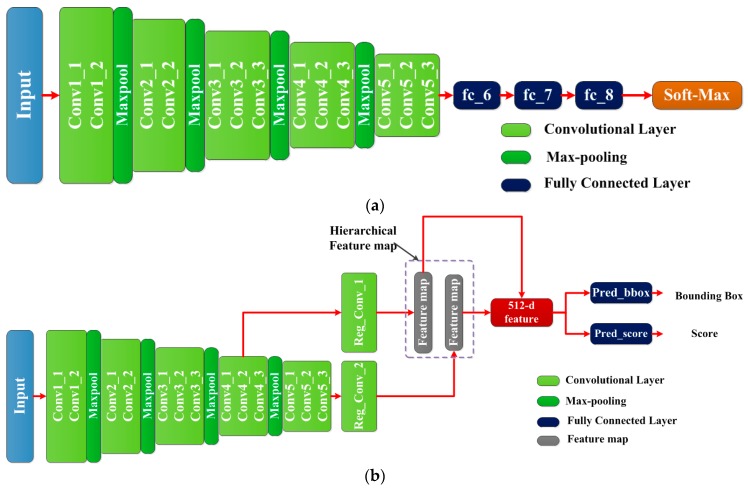
(**a**) The architecture of VGG-16 model; (**b**) The architecture of VPN.

The architecture of VPN is based on the VGG-16 model [21], which is a deep CNN including 13 convolutional layers and three fully connected layers (shown in Figure 4a). The original VGG-16 is an excellent model that is usually applied in image classification. Firstly, it generates a deep feature map by 13 convolutional layers. Then, the deep feature map is fed into the three fully connected layers to form a 4096-d (dimension) feature vector. Lastly, the feature vector is input into a soft-max for classification. However, VPN is used to deal with region-proposal task, which aims to not only predict the position of candidate regions, but also evaluate their objectness scores. Therefore, we reserve the 13 convolutional layers to generate multi feature maps, and make further modifications. Specifically, we modify this model by two strategies: (1) deleting the last three fully connected layers (from fc_6 to fc_8) and Soft-Max layer; (2) adding two small networks behind conv4_3 and conv5_3 respectively to generate candidate regions. The outputs of each small network are fed into two sibling fully connected layers for predicting bounding box and evaluating objectness score. Figure 4b illustrates the modifications and process of VPN.

Detailed descriptions of each layer are presented below:

Input data: this model requires RGB images (of any size) as the input.

Conv1 layers: Conv1 layers include two convolution layers (*conv1_1* and *conv1_2*), and the rectified linear units are configured after each convolutional layer. 64 kernels of sizes 3 × 3 are adopted for each layer.

Conv2 layers: configurations of Conv2 layers are almost as same as Conv1 layers’. The only difference is that Conv2 layers adopt 128 kernels of sizes 3 × 3.

Conv3, Conv4 and Conv5 layers include three convolutional layers, and the rectified linear units are configured after each convolutional layer. 256, 512 and 512 kernels (of size 3 × 3) are adopted respectively.

Pooling layers: this model adopts four pooling layers which are placed between the aforementioned Conv layers. The pooling layers are configured as max pooling with kernel of size 2 × 2.

Reg_Conv_1 layer and Reg_Conv_2 take *conv4_3* and *conv5_3* as the input respectively. Then, 512 kernels (of size 3 × 3) are adopted to generate two feature maps with different size.

Feature map: the hierarchical feature map architecture combines the output of the shallow convolutional layer and the deep convolutional layer. Because the shallower layers are better for localization and deeper layers are better for classification, the hierarchical feature map architecture integrates the advantages of both. Especially for small vehicles in aerial images, it shows better performance. In the hierarchical feature map architecture, a window of size 3 × 3 × 512 is slid to generate the vehicle-like regions. At each position, a 512-d (dimension) feature is extracted and fed into two sibling fully connected layers. The *pred_bbox layer* is used to predict the bounding box and the *pred_score layer* outputs a discrete probability distribution over two categories (vehicle-like region or background).

Following the anchor scheme in [19], this network predicts multiple regions associated with the different aspect ratios and scales at each sliding-window position. According to the average size of a vehicle (which is about 20 × 40 pixels), three aspect ratios (1:2, 1:1, 2:1) and four scales (16^2^, 32^2^, 48^2^, 64^2^) are set for vehicle-like regions. Hence, each sliding-window position generates 12 types of regions. We assign a positive label to the regions which have higher intersection-over-union (*IoU*) overlap ratio (which is greater than 0.7) with a ground-truth bounding box. Inversely, we assign a negative label to the regions which have lower *IoU* ratio (between 0.1 and 0.3) with ground-truth. The definition of *IoU* is seen as below (Equation (1)):

(1) $$IoU_{ratio}=\frac{A_{reg}\cap A_{gt}}{A_{reg}\cup A_{gt}}$$

where, *A_reg_* and *A_gt_* represent the bounding-box area of candidate regions and ground truth respectively.

#### 3.2.2. Loss Function

A multi-task loss function *L* (shown in Equation (2)) is employed to jointly train for classification and bounding-box regression:

(2) $$L(p^{t},l^{t})=L_{cls}(p^{t},p^{g})+\lambda *p^{g}*L_{br}(l^{t},l^{g})$$

For the *pred_score* layer, *p^t^* is the predicted probability of region being an object. The ground-truth label *p^g^* is 1 if the region is positive, and is 0 if the region is negative. *L_cls_* is log loss over two categories (vehicle-like region and background).

The *pred_bbox layer* outputs a vector representing the four parameterized coordinates (*x*, *y w*, *h*) of the predicted bounding box. *x*, *y*, *w*, and *h* denote the box’s center coordinates and its width and height. *l^g^* and *l^t^* represent the ground-truth bounding box and predicted bounding box respectively. And *L_br_* adopts smooth L1 loss function [18] defined in Equations (3) and (4). The parameter *λ* is the balancing parameter, and it is set to 10:

(3) $$\begin{array}{l} L_{br}(l^{t},l^{g})=S_{L1}(l^{t}-l^{g}) \end{array}$$

(4) $$S_{L1}(x)=\left\{\begin{array}{ll} 0.5x^{2} & \mathrm{if}\left|x\right|<1\\ \left|x\right|-0.5 & \mathrm{otherwise} \end{array}\right\}$$

#### 3.2.3. Training

The VPN is trained by the method of stochastic gradient descent (SGD) [45]. In the experiments, we initialize our model by a pre-trained VGG-16 weights which is previously trained on ILSVRC [14]. Because that the weights of new added convolutional layers should be initialized firstly, we initialize them by zero-mean Gaussian distribution with a 0.01 standard deviation, which is a widely used initialization way for CNN model in Caffe—deep learning framework [46]. Specifically, the initializations are configured in the model file (a ‘prototext’ file to describe the structure of the model). During training, a mini-batch is generated from one image, and it is set to 256. We keep the ratio of positive and negative examples to 1:1. If there are fewer than 128 positive examples in an image, we pad the mini-batch with negative ones. After the training process, VPN can generate a set of candidate regions; actually, there is no need to feed all of the regions to VDN. The works [10,11,12] have proven that top 2000 candidate regions almost cover all objects in the images. The RPN performs better than the traditional works [10] by adopting the top 300 candidate regions. As an improved version of RPN, the VPN also adopts top 300 highly overlapped candidate regions and feeds them into VDN for the further inference.

### 3.3. Vehicle Detection Network

Vehicle detection network takes the generated vehicle-like regions and image as the input and outputs a set of detections. The details of VDN are described as below.

#### 3.3.1. Overview of the Architecture

**Figure 5:**
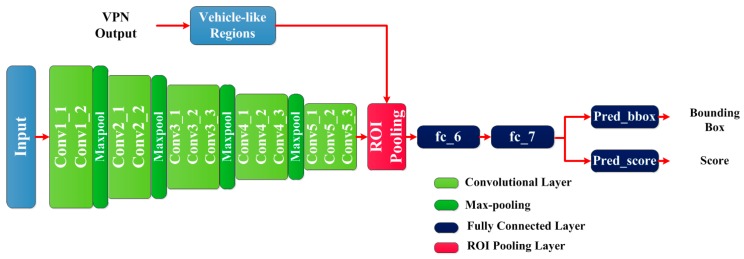
The architecture of VDN.

The architecture of VDN is also based on the VGG-16 model. Because the sizes and scales of the candidate regions are different, in order to extract the fixed-length feature vector from each region, the ROI polling layer [18] and two fully connected layers (fc_6 and fc_7) are adopted. Additionally, as a detection model, VDN is required to output the vehicle’s bounding box of and evaluate its confidence score. Two sibling fully connected layers are added behind fc_7 layer. Figure 5 illustrates the architecture of VDN.

Input data: this model requires two kinds of input data. One input is the same RBG image as the input of VPN. Another input is a set of candidate regions generated by VPN, which are directly mapped into ROI pooling layer.

Convolutional layers: the convolutional layers from Conv1 to Conv5 take identical settings as VPN.

ROI pooling layer: because the generated vehicle-like regions have various sizes, this layer extracts a fixed-length feature vector for each vehicle-like region. Specifically, this layer works by dividing the ROI (region of interest) window into a 6 × 6 grid of sub-windows and then max-pooling the values in each sub-window into the corresponding output grid cell [18]. Pooling is applied independently to each feature map channel, as in standard max pooling. The generated feature is the input of fc_6.

Fc_6 is a fully connected layer that outputs a 4096-d feature vector. Fc_7 takes the same settings as fc_6 and it is branched into two sibling fully connected layers, named *pred_bbox* and *pred_score* respectively. The *pred_bbox* layer predicts the bounding box of vehicle, and the output of *pred_score* layer is the corresponding confidence score.

#### 3.3.2. Loss Function and Training

The output of VDN and VPN is similar; therefore, the multi-task loss function *L* as given by Equation (2) is adopted to jointly train this network for vehicle classification and bounding-box regression. Moreover, the pre-trained VGG-16 weights are adopted as well. The training parameters and settings are similar to VPN.

## 4. Experiment and Results

We report the experimental results on two benchmark datasets: the VEDAI dataset [42] and the Munich vehicle dataset [43]. The performance of our detection model is compared with other methods on two aspects: detection accuracy and detection speed. Detailed evaluation metrics are described in Section 4.1. All methods in the experiments were programmed based on Matlab 2014a and Caffe deep learning framework [46]. All experiments were run on a desktop computer equipped with an Intel Core i7 5930k CPU (6 Core, 3.5 GHz), 64 GB memory, a NVIDIA Titan X GPU (with 12 GB video memory) and Ubuntu 14.04 OS.

### 4.1. Evaluation Metrics

We employ the widely used four metrics including: the precision-recall curve (PRC) [47], average precision (AP), recall rate and F1-Score [48] to quantitatively evaluate the performance of our model. The definition of F1-score is shown in Equation (5):

(5) $$F1\_Score=\frac{2*recall*precision}{recall+precision}$$

where, recall and precision are calculated by Equations (6) and (7):

(6) $$Recall=\frac{True\;Positive}{True\;Positive+False\;Negative}$$

(7) $$Precision=\frac{True\;Positive}{True\;Positive+False\;Positive}$$

Recall and precision evaluate the correctly identified positive detections and true positive detections respectively. The AP is defined as the area under the PRC, which is a comprehensive indicator of precision and recall rate. To sum up, F1-Score and AP are two key criteria to reveal the performance of detectors. The higher the F1-Score and AP score, the better the performance. In the experiments, the detections with *IoU_ratio_* value greater than 0.5 was defined as true, otherwise, it was false.

### 4.2. VEDAI Dataset

**Figure 6:**
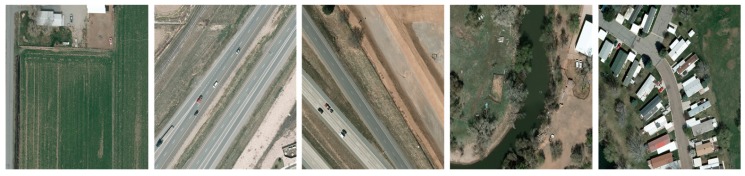
Examples from the VEDAI dataset.

VEDAI is a public dataset providing various types of vehicle in the images which were taken during spring 2012 in Utah, USA. The images comprise different backgrounds such as road, desert, rural and urban areas (shown in Figure 6). This dataset provides images with two different sizes, which are referred as VEDAI 512 (512 × 512 pixels) and VEDAI 1024 (1024 × 1024 pixels) respectively. VEDAI 1024 has a ground sampling distance of 12.5 cm/pixel, and the VEDAI 512 comprises the downscaled images of VEDAI 1024 and has a ground sampling distance of 25 cm/pixel.

This dataset contains nine different classes of vehicles, there are ‘car’, ‘pick-up’, truck’, ‘plane’, ‘boat’, ‘camping car’, ‘tractor’, ‘van’, and the ‘other’ category. There is an average of 5.5 vehicles per image, and they occupy about 0.7% of the total pixels of the images. The statistical data of each class is described in Table 1. Due to the scarcity of samples, we discard some categories (such as ‘boat’, ‘plane’ and ‘tractor’) in the experiments.

In the training stage, we adopted 996 images from VEDAI 1024 and augmented them according to the descriptions in Section 3.1. Each input image was resized such that its shorter side has 600 pixels. Moreover, for both networks (VPN and VDN), the training parameters were equivalent. We applied a weight decay of 0.0005 and a momentum of 0.9. There were 40,000 iterations in total during the whole training process, and the learning rate was set as 0.001 for the first 30,000 iterations, and 0.0001 for the next 10,000 iterations.

**Figure 7:**
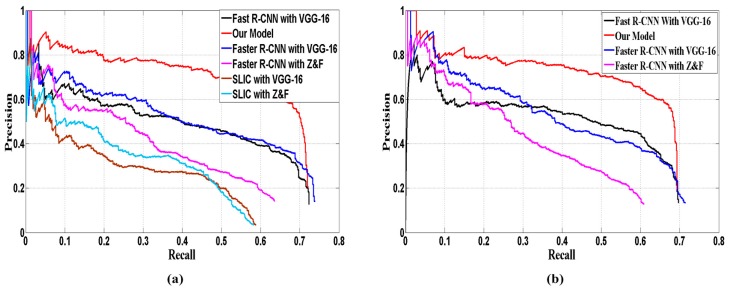
Precision-recall curve of four models: (**a**) VEDAI 1024 (**b**) VEDAI 512.

In the test stage, about 240 images (rest images of the dataset) with different size were selected to evaluate the performance. Our model was compared with super-pixels segmentation based methods (such as SLIC [29]) and recent CNN-based detectors, including: Faster R-CNN with Z&F model [49], Faster R-CNN with VGG-16 model and Fast R-CNN with VGG-16model. For the SLIC based methods, we first segmented the image into 768 regions by SLIC, and then converted the generated polygonal regions into approximate rectangular regions. The converted regions were fed into VGG-16 and Z&F model respectively. These two models were referred as: SLIC with VGG-16 and SLIC with Z&F. As the comparison results in Table 2 illustrate, for VEDAI 1024, our detection model outperforms the super-pixels segmentation based methods and recent CNN-based detectors, which obtains the best AP (54.6%) and F1-score (0.305). Especially, the AP outperforms the second best detector by 12.5 percentage points. And the recall rate also reaches a comparable level with Faster R-CNN (VGG 16). For VEDAI 512, our model obtains the best AP and F1-Score as well. Figure 7a,b show the PRC of the various models on VEDAI 1024 and VEDAI 512, respectively. Compared with other models, our model shows significant improvement.

**Figure 8:**
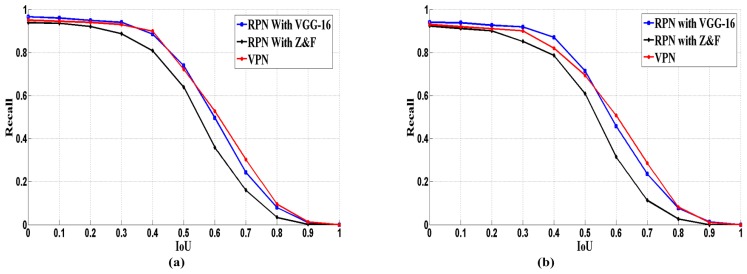
Recall vs. *IoU* curve of three CNN-based models: (**a**) VEDAI 1024 (**b**) VEDAI 512.

The performance of VPN determines the results of detection model, to evaluate the localization performance of VPN; we compared it with other RPN-based region proposal methods. Reference [19] designed the RPN based on Z&F and VGG-16 model respectively. We adopted the recall-IoU curve (shown in Figure 8) for evaluation.

As the results in Figure 8 show, our model obtains a comparable recall rate to Faster R-CNN (with VGG-16). When the *IoU_ratio_* is greater than 0.5, our model achieves the best performance. Additionally, we evaluated the detection speed of different detection models by *fps* (frames per second). Table 3 illustrates the detection time and training time of each detection model. From the aspect of detection time, our model, SLIC based models and other two Faster R-CNNs achieve comparable detection speed. The Fast R-CNN that uses the Selective Search [10] scheme for region proposal performs poorly, and its detection speed is much slower than the speed of the other five. The Faster R-CNN (with Z&F model) adopts a simple and shallow CNN, so it achieves the fastest detection speed. However, it obtains the lower detection accuracy (30.8% and 32%). The SLIC based models perform well on detection speed, which are benefit for the segmentation speed of SLIC algorithm, but they obtain the lowest detection accuracy (23.2%). This may be caused by the inaccurate segmentation and the conversion of segmented regions. The detection speed of our model is a little slower than Faster R-CNN with VGG-16, because the proposed VPN is a hierarchy architecture, which spends a little more time on generating more but accurate candidate regions. Actually, this gap is very small in practical application. Hence, we made the trade-off between detection speed and accuracy. For the training time, Fast RCNN and SLIC based models perform well, because training CNN is time consuming and they just adopt one CNN for feature extraction, the rest models employ two CNNs for region proposal and feature extraction respectively. Our model is better than the Faster RCNNs, because the Faster RCNNs are alternatively trained twice, but we train each CNN (VPN and VDN) only once. In practical application, detection time is considered more. Due to the fact that detection systems always adopt the trained model and no extra training cost during the detection phase.

**Figure 9:**
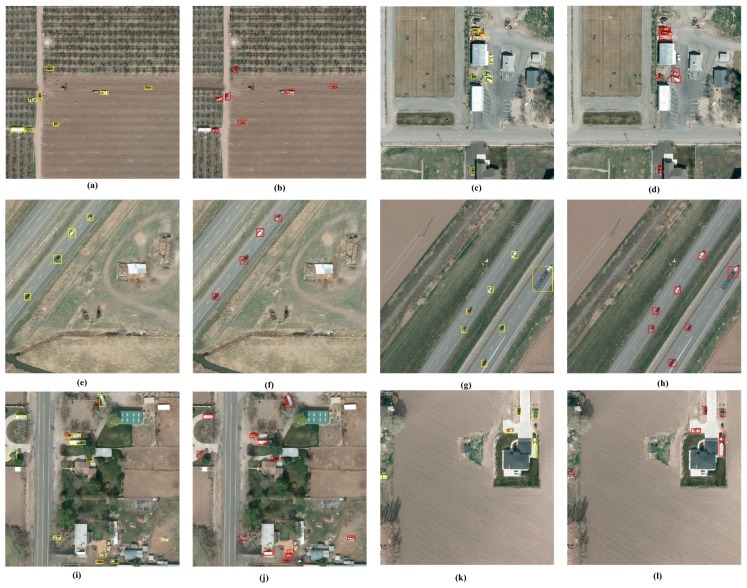
(**a**–**l**) some detection examples of VEDAI dataset.

Figure 9 shows some detection examples of VEDAI 1024. Figure 9a,c,e,g,i,k is the input images, and the ground truths are annotated by yellow boxes. Figure 9b,d,f,h,j,l is the detections annotated by red boxes.

### 4.3. Munich Vehicle Dataset

The Munich vehicle dataset is an aerial imagery dataset captured by the DLR 3 K camera system [50] over the area of Munich, Germany. It comprises of 20 aerial images which were mainly taken from urban and residential areas. The original images in this dataset were taken at the height of 1 km above the ground with the resolution of 5616 × 3744 pixels, and the approximate ground sampling distance is 13 cm/pixel. Training and testing set include 10 images respectively.

We performed our model on the testing set and compared the performance with other two RPN-based models (Faster R-CNN with VGG-16 and Faster R-CNN with Z&F). In the training process, we firstly cropped the original images into the size of 702 × 468; in this way, then collected 640 training images from Munich dataset. Secondly, we combined the training set of VEDAI 1024 and these cropped images to form a joint training set. During training, we used the same parameters and settings as that were adopted in VEDAI dataset.

In testing phase, each testing image was cropped into 702 × 468 pixels as well. Hence, 640 cropped images were employed as the testing set. As the evaluation results showed in Table 4, our model obtains the best detection accuracy. Especially, the AP outperforms other two models by approximate 20 and 10 percentage points. The detection speed also achieves a comparable level with that of others.

**Figure 10:**
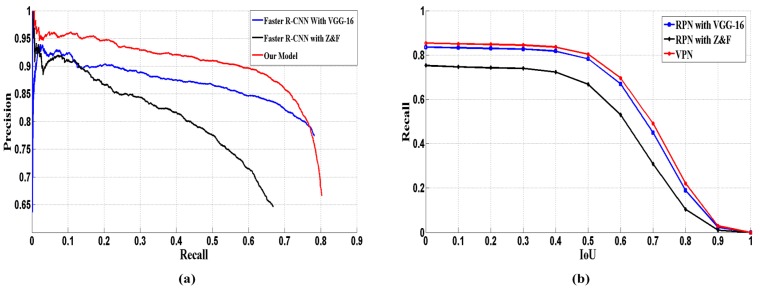
Comparisons of three detection models (**a**) precision-recall curve (**b**) recall vs. IoU curve.

**Figure 11:**
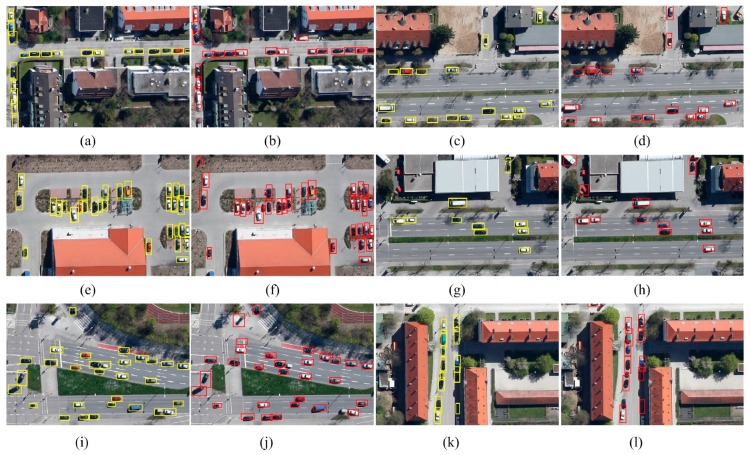
(**a**–**l**) some detection examples of VEDAI dataset.

In addition, the precision-recall curve and recall-IoU curve are showed in Figure 10a,b. Figure 11 gives some examples of the detection on the Munich vehicle dataset. Figure 11a,c,e,g,i,k is the input images, and the ground truths are annotated by yellow boxes. Figure 11b,d,f,h,j,l is the detect results, and the detections are annotated by red boxes.

## 5. Conclusions

In this paper, we propose a fast and accurate vehicle detection model for aerial images. Unlike the traditional sliding-window-based detection models and recent CNN-based models, our detector is a cascaded CNNs architecture that combines two CNNs (VPN and VDN) for generating candidate regions and making decisions, respectively. The proposed VPN is based on a VGG-16 model; taking advantage of the shallow and deep feature map, we build hierarchical feature maps. Compared with other CNN-based region proposal methods (such as RPN with VGG-16, RPN with Z&F), the VPN generates more accurate candidate regions, especially for the small vehicles in aerial images. Moreover, we trained a category-specific detection network called VDN, which is combined with VPN and obtained high performance. From the extensive experimental results presented in Section 4, the proposed model outperforms the state-of-the-art detection model [18,19] in detection accuracy, and the detection speed achieves a comparable level.

Although our model has obtained favorable performance on vehicle detection in aerial image data, it still has some limitations. One limitation is in hard example detection, for example, when some vehicles in aerial images are partially occluded by other objects or extremely small vehicles. Moreover, to distinguish some intra-class vehicles is also difficult, such as camping cars and big vans. In the future work, we focus on the further optimization of VPN. Firstly, a deeper CNN model will be adopted and built finer architecture of feature maps. Moreover, to reduce the time cost of region proposal stage, we will try to improve the performance of the efficient super-pixel segmentation method like SLIC, which shows advantages in speed of generating regions, but the capability of generating accurate candidate regions should be improved. Multi-GPUs should be adopted collaboratively in the region proposal stage.

## Figures and Tables

**Table 1:** The statistical data of VEDAI.

Classes	Tag	Number
Car	car	1340
Pick-up	pic	950
Truck	tru	300
Plane	pla	47
Boat	boa	170
Camping car	cam	390
Tractor	tra	190
Vans	van	100
Other	oth	200

**Table 2:** Comparison results of various detection models on VEDAI.

Detection Model	Image Size	Recall Rate	AP	F1-Score
Faster R-CNN (Z&F)	1024 × 1024	63.5%	30.8%	0.229
Faster R-CNN (VGG-16)	1024 × 1024	**73.9%**	42.1%	0.232
Fast R-CNN (VGG-16)	1024 × 1024	72.2%	39.8%	0.216
SLIC with Z&F	1024 × 1024	58.3%	25.4%	0.066
SLIC with VGG-16	1024 × 1024	58.8%	23.2%	0.064
Our Model	1024 × 1024	72.3%	**54.6%**	**0.320**
Faster R-CNN (Z&F)	512 × 512	60.9%	32.0%	0.212
Faster R-CNN (VGG-16)	512 × 512	**71.4%**	40.9%	0.225
Fast R-CNN (VGG-16)	512 × 512	69.4%	37.3%	0.224
Our Model	512 × 512	69.7%	**50.2%**	**0.305**

**Table 3:** Comparison of detection time (fps: frames per second) and training time (h: hours).

Detection Model	Image Size	Detection Time	Training Time
Faster R-CNN (Z&F)	1024 × 1024	**5.8 fps**	28.4 h
Faster R-CNN (VGG-16)	1024 × 1024	5.4 fps	28.5 h
Fast R-CNN (VGG-16)	1024 × 1024	0.4 fps	8.2 h
SLIC with Z&F	1024 × 1024	5.6 fps	7.9 h
SLIC with VGG-16	1024 × 1024	4.9 fps	8.2 h
Our Model	1024 × 1024	4.5 fps	10.7 h
Faster R-CNN (Z&F)	512 × 512	**6.3 fps**	28.3 h
Faster R-CNN (VGG-16)	512 × 512	5.6 fps	28.6 h
Fast R-CNN (VGG-16)	512 × 512	0.4 fps	8.1 h
Our Model	512 × 512	4.6 fps	10.6 h

**Table 4:** Comparison results of various detection models on Munich Vehicle dataset.

Detection Model	Recall Rate	AP	F1-Score	Detection Time (fps)
Faster R-CNN (Z&F)	66.8%	53.9%	0.657	**5.2**
Faster R-CNN (VGG-16)	78.3%	64.8%	0.779	4.9
Our Model	**80.3%**	**73.7%**	**0.782**	3.2
